# Vitamin A Absorption Efficiency Determined by Compartmental Analysis of Postprandial Plasma Retinyl Ester Kinetics in Theoretical Humans

**DOI:** 10.1093/jn/nxaa176

**Published:** 2020-07-02

**Authors:** Michael H Green, Joanne Balmer Green, Jennifer Lynn Ford

**Affiliations:** Department of Nutritional Sciences, College of Health and Human Development, The Pennsylvania State University, University Park, PA, USA; Department of Nutritional Sciences, College of Health and Human Development, The Pennsylvania State University, University Park, PA, USA; Department of Nutritional Sciences, College of Health and Human Development, The Pennsylvania State University, University Park, PA, USA

**Keywords:** vitamin A, absorption, chylomicrons, retinyl esters, humans, WinSAAM

## Abstract

**Background:**

Better methods are needed for determining vitamin A absorption efficiency in humans to support development of dietary recommendations and to improve the accuracy of predictions of vitamin A status.

**Objectives:**

We developed and evaluated a method for estimating vitamin A absorption efficiency based on compartmental modeling of theoretical data on postprandial plasma retinyl ester (RE) kinetics.

**Methods:**

We generated data on plasma RE and retinol kinetics (30 min to 8 h or 56 d, respectively) after oral administration of labeled vitamin A for 12 theoretical adults with a range of values assigned for vitamin A absorption (55–90%); we modeled all data to obtain best-fit values for absorption and other parameters using Simulation, Analysis, and Modeling software. We then modeled RE data only (16 or 10 samples), with or without added random error, and compared assigned to predicted absorption values. We also compared assigned values to areas under RE response curves (RE AUCs).

**Results:**

We confirmed that a unique value for vitamin A absorption cannot be identified by modeling plasma retinol tracer kinetics. However, when RE data were modeled, predicted vitamin A absorptions were within 1% of assigned values using data without error and within 12% when 5% error was included. When the sample number was reduced, predictions were still within 13% for 10 of the 12 subjects and within 23% overall. Assigned values for absorption were not correlated with RE AUC (*P* = 0.21).

**Conclusions:**

We describe a feasible and accurate method for determining vitamin A absorption efficiency that is based on compartmental modeling of plasma RE kinetic data collected for 8 h after a test meal. This approach can be used in a clinical setting after fasting subjects consume a fat-containing breakfast meal with a known amount of vitamin A or a stable isotope label.

## Introduction

Although many aspects of whole-body vitamin A metabolism have been actively studied over the past decades, vitamin A absorption has not been adequately quantified (1, 2). Thus, researchers must make assumptions about absorption efficiency when developing recommendations for vitamin A intake, estimating vitamin A status using retinol isotope dilution (RID), or applying model-based compartmental analysis to make predictions about whole-body vitamin A metabolism and stores.

Several approaches have been explored for estimating vitamin A absorption efficiency in humans and animal models. These include direct measurement of labeled vitamin A in lymph of cannulated rats (3) or humans (4), indirect assessment based on recovery of label in feces after ingestion of an oral dose of labeled vitamin A by children (5, 6), and determination of the isotope ratio in plasma after administration of 1 label orally and another intravenously to rats (7). In addition, area under the curve (AUC) methods have been used to estimate vitamin A and carotenoid bioavailability (8–12). None of these approaches have been extensively applied, thus limiting the data available and researchers’ confidence in it.

Given the unique capacity of model-based compartmental analysis to quantify other aspects of the vitamin A system (13), one might hypothesize that it would be an ideal technique for estimating vitamin A absorption efficiency; to our knowledge, this type of modeling has not yet been used for such purposes. In a typical human whole-body retinol kinetic study, a stable isotope of vitamin A is administered orally and serial blood samples are obtained for various periods of time; the retinol tracer response in plasma is then analyzed in light of a compartmental model that describes whole-body vitamin A metabolism, from absorption through utilization. Although most processes are well defined using this approach, it was surprising to find [MJ Haskell (University of California, Davis) and MH Green, unpublished results 1997] that, for 1 data set, plasma retinol kinetic data could be equally well fitted assuming a wide range in absorption efficiencies (40–80%). These results indicate that data on plasma retinol tracer response versus time after administration of labeled vitamin A are not sufficient to accurately determine absorption. However, it is likely that including early data on plasma chylomicron retinyl ester (RE) kinetics would enable definition of the extent of vitamin A absorption.

Here, we tested and confirmed the hypothesis that one can accurately determine vitamin A absorption efficiency by applying model-based compartmental analysis to postprandial plasma RE response data. By using a data set based on theoretical humans, as was done in other studies (14–18), we were able to compare model predictions to assigned values for vitamin A absorption efficiency and thereby validate our method.

## Methods

### Generation of theoretical data

**FIGURE 1:**
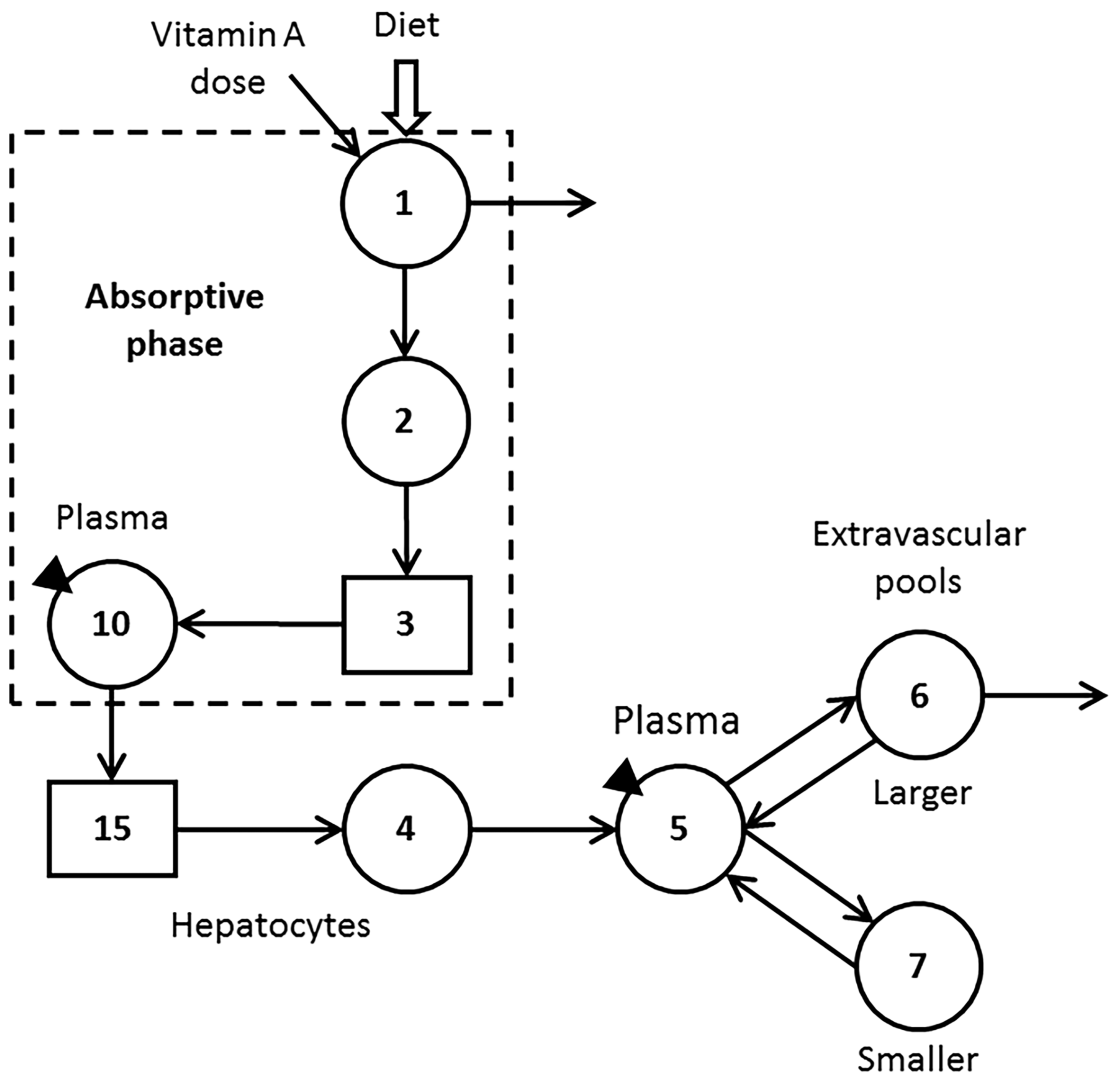
Proposed compartmental model for whole-body vitamin A kinetics in humans. Circles represent compartments; the rectangles are delay elements; interconnectivities between components (arrows) are fractional transfer coefficients [L)I,J)s, or the fraction of retinol in compartment J transferred to compartment 1 each day]; and DT(I)s are delay times (or the time spent in delay element I). Components 1–3 represent the processes of vitamin A digestion and absorption plus the production of chylomicrons; compartment 1 is the site of introduction of the vitamin A dose and dietary vitamin A as well as the site of loss for unabsorbed vitamin A; L(2,1) was fixed at 30 d^−1^ to reflect transit through the upper gastrointestinal tract. Chylomicrons are metabolized in plasma compartment 10 before hepatocyte uptake of retinyl esters in chylomicron remnants (delay element 15 and compartment 4). Retinol bound to retinol-binding protein is secreted from compartment 4 into plasma compartment 5; retinol in plasma can exchange with vitamin A in 2 extravascular pools (a larger compartment 6 and a smaller compartment 7); compartment 6 is the site of irreversible loss from the system. The triangles indicate that plasma is the site of sampling (compartment 10 for retinyl esters and compartment 5 for retinol).

Using an approach similar to that presented by Ford et al. (18), we hypothesized 12 theoretical adults, assigning a range of values (**Supplemental Table 1**) for vitamin A absorption efficiency, kinetic parameters, and state variables including vitamin A total body stores (TBS). Parameters and state variables were assigned for each subject based on published values (18–20); specifically, we designated a range for each parameter and then randomly assigned a value within that range for each subject, except that subjects with higher vitamin A absorption efficiencies were assigned values for TBS that were at the upper end of the range and vice versa. Parameters were assigned in light of a previously published (21) 7-component compartmental model describing whole-body vitamin A metabolism that we adapted to include 2 components related to vitamin A absorption: compartment 10 to represent chylomicron REs and component 15 to represent chylomicron remnant processing in hepatocytes (**Figure 1**). We assumed that fasting subjects consumed an oral dose of stable isotope-labeled retinyl acetate at time-zero (*t*_0_). We then used WinSAAM [the Windows version of the Simulation, Analysis, and Modeling software; www.WinSAAM.org; (22–24)] to simulate fraction of dose data for plasma chylomicron REs (compartment 10, Figure 1) from 0.5 to 8 h (16 samples in 30 min intervals) and for plasma retinol bound to retinol-binding protein (compartment 5) from 30 min to 56 d (29 samples).

### Modeling of theoretical data

First, we used WinSAAM to find a best fit to the simulated retinol and RE data for each subject in order to confirm that the model accurately predicted the assigned values for vitamin A absorption, kinetic parameters, and state variables. Second, to more systematically explore our observation (see Introduction) that plasma retinol tracer data versus time are not sufficient to determine a unique value for vitamin A absorption, we modeled retinol data versus time for each subject, assuming an absorption efficiency of 75%, the value we have used in previous studies in adults (18, 19, 25); we then compared model-predicted vitamin A absorption efficiencies and TBS with the assigned values.

Next, we modeled only the RE data in light of the absorptive phase of the model (compartments 1–3 and 10; dashed box in Figure 1) and compared model-predicted vitamin A absorptions with the assigned values. Then, to better mimic real observed data, we used WinSAAM's RAND command to add error with a normal distribution and a fractional SD of 5% to the simulated data for compartment 10 (“RE data with error”); see **Supplemental WinSAAM Deck**. Note that each use of the RAND command produces a unique set of simulated data. For each subject, we modeled the RE data with error and compared model-predicted vitamin A absorptions to the assigned values. Finally, we reduced the number of RE sampling times from 16 to 10 (0.5, 1, 1.5, and 2–8 h), simulating an 8-h clinical study; we then repeated the modeling and compared predicted absorptions to the assigned values.

We also asked whether vitamin A absorption could be determined by this method but without the use of a tracer. To investigate this idea, we assumed that subjects were fasted overnight to clear absorption-related compartments (compartments 1–3 and 10; dashed box in Figure 1) of vitamin A, a *t*_0_ blood sample was obtained to measure baseline plasma RE concentrations, and a breakfast meal containing the RDA for vitamin A was consumed. We used WinSAAM to follow the mass response of vitamin A in compartment 10 (chylomicron REs) as fraction of vitamin A in the meal, fitting the simulated data to the model to determine absorption efficiency. We then compared model predictions to the assigned values for absorption for each subject.

### Correlation of vitamin A absorption efficiency and area under the plasma RE tracer response curves

We calculated RE AUC from 0 to 8 h for each subject and then determined the correlation between AUC and the assigned values for vitamin A absorption efficiency.

### Data management and statistics

Data are presented as individual values and, when relevant, means or ranges are also presented. Figures were created using Microsoft PowerPoint and Adobe Photoshop or with Prism (v. 7.0; GraphPad). Predicted values for vitamin A absorption were compared with assigned values using standard least squares ANOVA (with “method” as the effect) in JMP Pro (v. 13; SAS Institute Inc.). Areas under the plasma RE response curves were integrated using the trapezoidal rule in Prism software; correlations between AUCs and assigned values for vitamin A absorption were evaluated using Prism's least squares regression analysis. *P* <0.05 was considered significant.

## Results

### Theoretical data

As shown in Supplemental Table 1, assigned values for the 12 theoretical adult subjects ranged from 55 to 90% for vitamin A absorption and from 160 to1800 μmol for TBS (sum of mass of vitamin A in compartments 6 and 7; Figure 1); plasma retinol pool size was set at 5 μmol for all individuals, reflecting normal vitamin A status (19). Assigned values for kinetic parameters are also listed in the table.

### Data modeling and predicted vitamin A absorption efficiencies

When simulated data for plasma retinol (0 to 56 d) and REs (0 to 8 h) were fitted to the model to obtain best-fit values, there was excellent agreement with the assigned values for both vitamin A absorption efficiency and TBS (within 1 and 6%, respectively; data not shown). These results confirm that the WinSAAM code and fitting procedure worked as expected for an extensive plasma retinol and RE data set.

**FIGURE 2:**
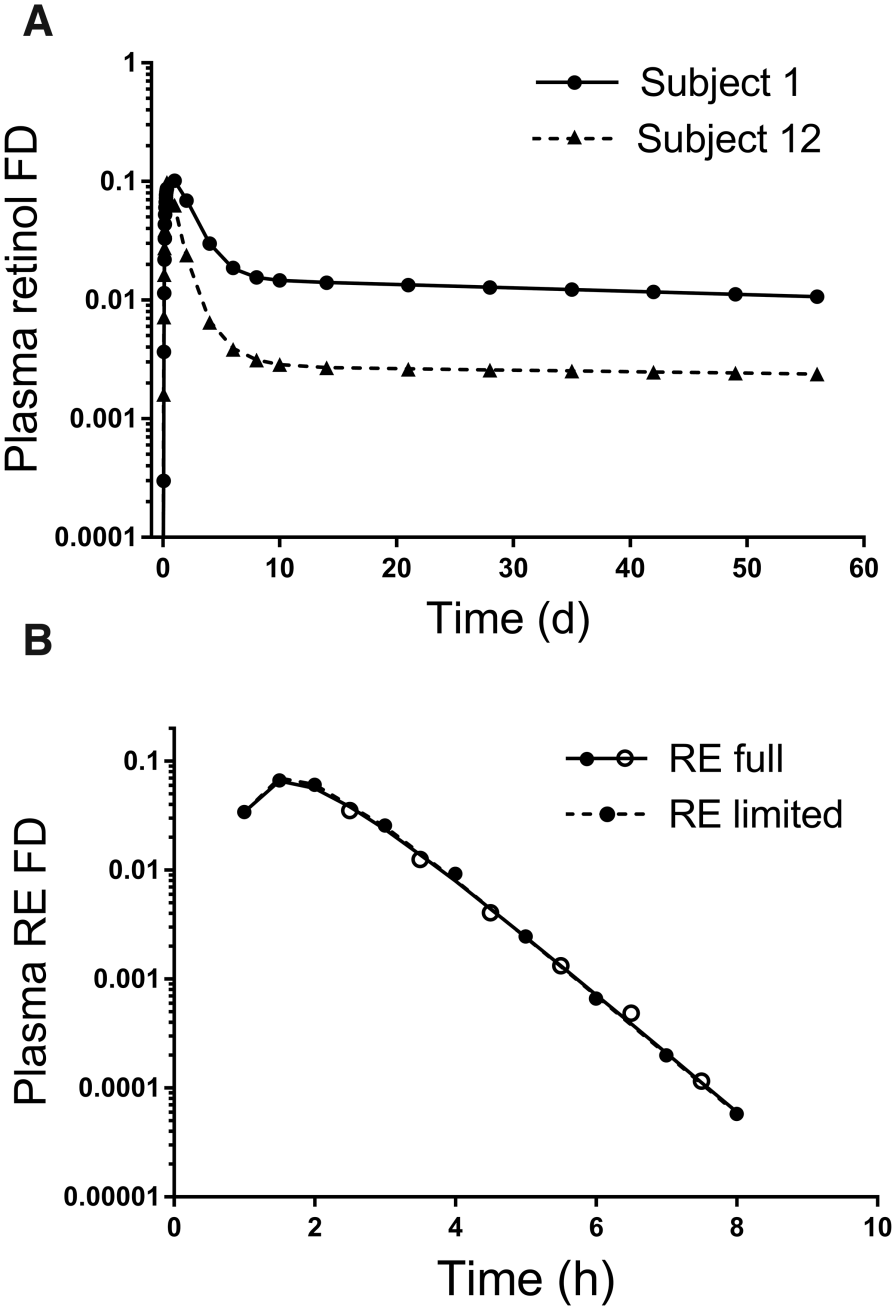
Simulated data (symbols) and model predictions (lines) for plasma retinol over 56 d for subjects 1 and 12 (A) and for retinyl esters over 10 h for subject 6 (B). In panel A, plasma retinol data (without error) as fraction of the oral dose are shown for subjects who had assigned values for vitamin A absorption efficiency of 55 (subject 1) and 90% (subject 12). Absorption was fixed at 75% to obtain the model predictions. In panel B, data are plasma REs with 5% random error for the subject whose assigned value for vitamin A absorption efficiency was the same as the mean for the group (72%) and model-calculated fits to the full (16 samples) and limited sampling data sets (10 samples). See Supplemental Table 1 for subject details. FD, fraction of administered dose; RE, retinyl ester.

To further investigate our observation (see Introduction) that plasma retinol tracer data alone are not sufficient to determine a unique value for vitamin A absorption, we assumed, as has been done in our previous retinol modeling studies in adults (18, 19, 25), that absorption efficiency was 75% and then we modeled each individual's plasma retinol data. **Figure 2**A shows simulated data for subjects 1 and 12, who had the lowest (55%) and highest assigned values (90%), respectively, for vitamin A absorption efficiency and values of 159 and 1448 μmol for TBS. Also shown are the final model-calculated fits to the data with absorption efficiency fixed at 75%. Fits to the data for these 2 subjects were excellent, based on residual sums of squares and the ratio of calculated to observed data at each sampling time; the same was true for other subjects (data not shown). These results indicate that, for all 12 theoretical subjects, we could fit the plasma retinol tracer data accurately using a value of 75% absorption, in spite of the fact that assigned values for absorption ranged from 55 to 90%; the ratio (75%/assigned absorption) ranged from 1.36 when assigned absorption was 55% to 0.83 when it was 90%. Analogous results were observed for the ratio of TBS predicted when absorption was set at 75% compared with the assigned TBS (range, 1.38 to 0.81). That is, the error in model-predicted TBS was proportional to the error in absorption efficiency.

Next, we modeled the simulated RE data in light of the absorptive phase of the model (compartments 1–3 and 10; dashed box in Figure 1). We first focused on the RE data without error and a robust sampling schedule (*n* = 16), then the RE data with 5% random error and the same sampling times, and finally, the RE data with 5% error and a reduced number of samples (*n* = 10). Shown in Figure 2B is the tracer response curve for subject 6 (using RE data with 5% error), whose assigned value for vitamin A absorption efficiency was 72%, the mean for the group. Plasma RE fraction of dose peaked at 1.5 h for this subject and then declined rapidly over the ensuing 6.5 h, reflecting clearance of REs from chylomicron remnants; qualitatively similar curves were obtained for other subjects (data not shown but plots can be simulated using parameters presented in Supplemental Table 1). When we compared the assigned absorption values for each subject to the predictions obtained by modeling the 3 RE data sets, we found excellent agreement between predicted and assigned values (**Table 1**); mean predictions among the 3 RE data sets were not significantly different. Specifically, for all subjects, using the full RE data set without error, predicted absorption was within 1% of the assigned values. Even when a normally distributed error of 5% was added to the RE data, predictions of vitamin A absorption were still within 12% of the assigned values over all subjects. When the number of RE sampling times was reduced to 10 (0, 1, 1.5, and 2–8 h), those data were adequately sensitive to predict the assigned absorption values to within a mean of 3% (within 13% for 10 of the subjects and to within 23 and 20% for the 2 others).

**TABLE 1:** Assigned and model-predicted vitamin A absorption efficiencies for theoretical subjects^1^

		Predicted		
ID	Assigned	RE model	RE 5% error	Limited RE 5% error
S1	55	55	57	61
S2	59	59	62	67
S3	64	64	60	59
S4	67	67	72	71
S5	70	72	62	54
S6	72	72	64	58
S7	74	74	74	73
S8	75	75	74	75
S9	77	77	78	71
S10	80	80	80	77
S11	86	88	86	82
S12	90	90	90	89
Mean	72	73	72	70

1 Shown are assigned values and the mean for vitamin A absorption efficiency for 12 theoretical adults as well as values predicted by modeling RE kinetics from 30 min to 8 h (16 samples) after oral administration of labeled retinyl acetate (“RE model”), the same data sets with 5% random error (“RE 5% error”), and a limited RE data set (10 samples) with 5% random error (“limited RE 5% error”). There were no significant differences in assigned versus predicted absorption efficiencies among the data sets based on standard least squares ANOVA and Tukey honest significant difference test. See Supplemental Table 1 for additional subject details. RE, retinyl ester.

Values for the adjustable parameters describing vitamin A absorption [L(0,1), DT(3), and L(15,10)] obtained from modeling the 3 RE data sets are presented in **Supplemental Table 2** along with the assigned values. Note that the terminal slope for the RE curve is L(2,1), not L(15,10), since the former rate constant is the rate-limiting step in the absorption process.

### Prediction of vitamin A absorption without the use of a label

Since chylomicrons are not present in plasma in the fasting state and only transiently in the postprandial state in normolipidemic individuals who have normal vitamin A status, we reasoned that the method proposed here might not require use of a vitamin A tracer. Indeed, by modeling simulated data for unlabeled REs versus time after fasting subjects consumed a fat-containing breakfast meal with a known amount of vitamin A and expressing the data as fraction of dose as we did for the tracer, one obtains the same model-predicted vitamin A absorption efficiencies in these theoretical subjects as when a tracer was included in the meal (Table 1). That is, the results of the analyses presented here are the same for a tracer or a tracee study.

### Correlation between assigned values for vitamin A absorption and area under the plasma RE response curves

**FIGURE 3:**
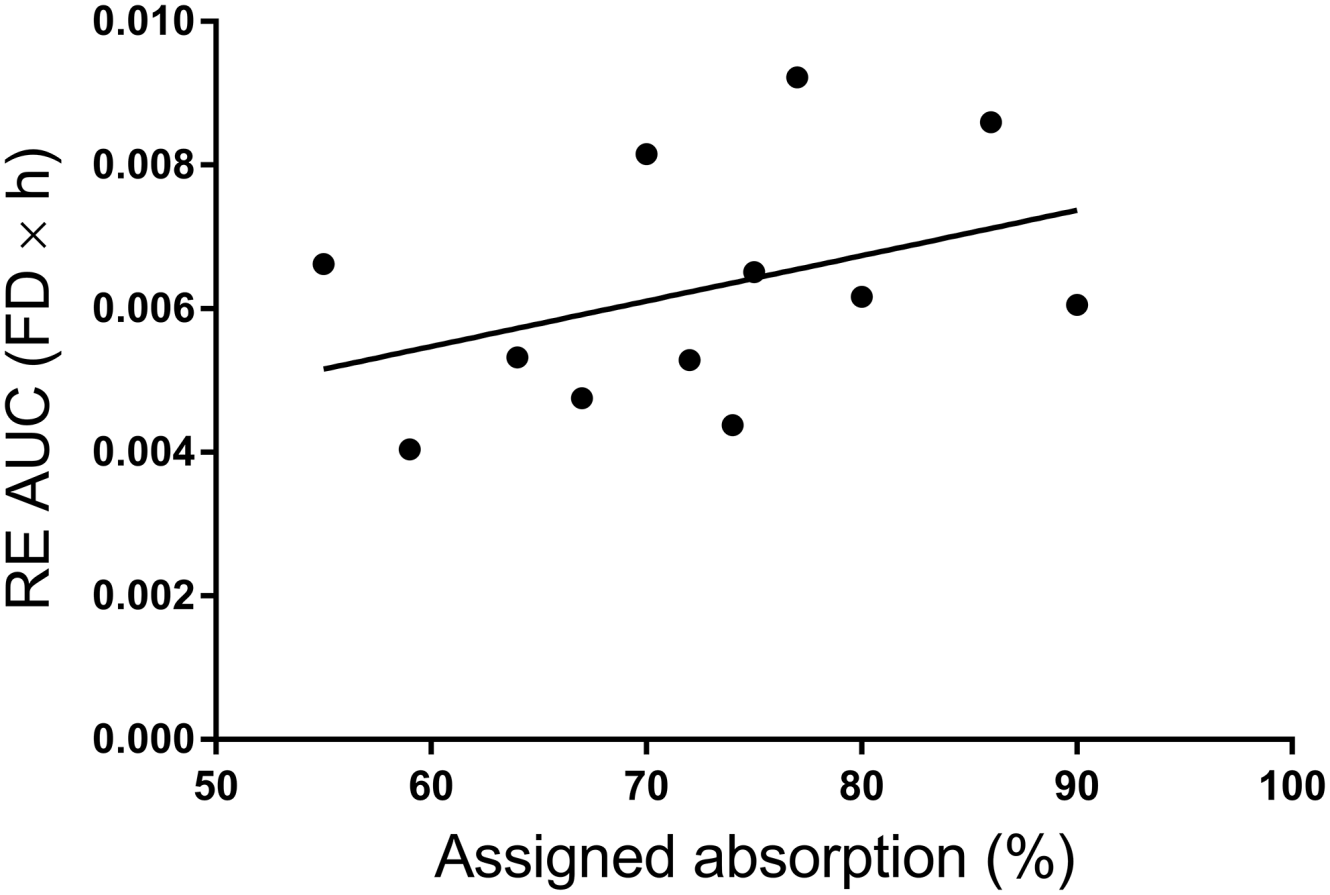
Assigned vitamin A absorption versus area under the RE tracer response curve over 8 h for 12 theoretical subjects. AUCs were calculated using Prism software. Data fit the equation *Y* = 0.000063X + 0.0017; *R*^2^ = 0.153 (*P* = 0.21; NS). FD, fraction of administered dose; RE, retinyl ester.

As shown in **Figure 3**, there was no significant correlation between assigned values for vitamin A absorption efficiency and RE AUCs (*P* = 0.21).

## Discussion

Here, we propose and test a method for measuring vitamin A absorption efficiency in humans based on compartmental analysis of plasma RE kinetics after subjects ingest a vitamin A-containing meal (with or without a stable isotope tracer). This approach requires serial blood sampling (a minimum of 10 samples over at least an 8-h period) and thus a clinical setting; however, it has advantages over other techniques that have been applied to estimate vitamin A absorption. For example, fecal recovery of unabsorbed label has been successfully used in children (6), but it requires complete stool collection and may be impacted by bacterial degradation of label and analytical difficulties. A plasma isotope ratio method has been applied in rats (7), but this technique may be challenging to replicate in humans because of the costs and safety precautions related to the need for intravenous administration of 1 of the labeled vitamin A doses. As shown here (Table 1), our new method provided accurate estimates of vitamin A absorption efficiency over a wide range (55–90%) in theoretical humans. If both this test and the plasma isotope ratio method could be tested in humans, a comparison of the results would be highly informative. Our current results also demonstrate that absorption can be measured without the use of a label, which would allow analyses to be performed using ultra performance liquid chromatography instead of the LC/MS/MS required for stable isotope studies.

If our method were to be used in humans in a clinical setting, several points would need to be considered. To enable the study to be completed in an 8-h period, the breakfast meal should have adequate satiety value so that subjects are comfortable during the initial hours of the test. Importantly, the breakfast meal needs to contain sufficient fat (∼10 g) to maximize vitamin A absorption but not so much that chylomicron REs peak in plasma >4 h after consumption of the meal (9). With a modest fat load, the early sampling schedule (30 min intervals for the first 4 h) ensures that the RE peak will be adequately captured in the initial samples so that data are optimally sensitive for compartmental modeling. If the fat load is too high, vitamin A absorption will be slower and stretched over a longer period of time (12) and thus the proposed sampling schedule may not be sufficient to describe absorption of the test dose in an 8-h study. Also, the vitamin A load must be large enough to be quantifiable but reasonable enough that essentially all the absorption occurs before lunch, thus minimizing subsequent output of label when the next meal is consumed. The breakfast meal could be vitamin A free, with the test dose provided via a supplement for which vitamin A content has been measured.

The transient nature of absorptive lipoprotein REs in plasma makes it possible to apply this method to either tracer or tracee data. In the case of modeling plasma retinol kinetics, a tracer is required to follow the temporal response because retinol concentrations are maintained in a steady state. In contrast, since basal concentrations of RE in plasma VLDLs are low in individuals with normal vitamin A status, the spike in RE concentrations after a meal can be monitored without the use of a tracer. That is, in the postabsorptive state (e.g., after an overnight fast), subjects would consume a fat-containing breakfast meal with a known amount of unlabeled vitamin A and the resulting RE responses would be analyzed in light of the absorptive phase of the model shown in Figure 1 (dashed box) to predict vitamin A absorption efficiency. Eliminating the need for a stable isotope label would both decrease costs and simplify the analyses required when applying this method.

As part of this work, we also demonstrated (Figure 3) that plasma RE AUC is not a proxy for vitamin A absorption efficiency even though AUCs have been previously used to estimate vitamin A and β-carotene bioavailability (8–12). Here, since RE AUC is equivalent to the mean residence time that REs entering the system at compartment 1 (Figure 1) spend in plasma and because that time is a function of both absorption efficiency and the chylomicron RE fractional catabolic rate [L(15,10)], it is not surprising that the 2 parameters are not highly correlated. That is, due to interindividual variability in absorption efficiency and plasma chylomicron clearance rates, it should not be concluded that AUC is directly related only to absorption.

As noted in the Introduction, there are several important areas of research in the vitamin A field that assume an accurate estimate of absorption. In the case of the application of RID to assess vitamin A status, vitamin A absorption is a key factor in prediction equations (26–28). In fact, estimates of vitamin A TBS obtained by RID (or by compartmental modeling) will be incorrect in proportion to the difference between assumed and actual vitamin A absorption. For example, if absorption is assumed to be 90% but is actually 75%, TBS predicted by RID will be 20% higher than the actual value. These results emphasize the need for investigators to have estimates of vitamin A absorption that are as accurate as possible. Our current results (Table 1) indicate that the modeling of short-term RE kinetics in plasma provided estimates that were within 12% of the assigned value for these 12 theoretical adult subjects using the extensive sampling protocol (16 samples) and within 13% for 10 of the 12 subjects using the reduced sampling schedule (10 samples).

Our new method could be included as an enhancement to future population-based (“super-person”) vitamin A kinetic studies, an approach that has been used to study retinol kinetics in children in whom extensive blood sampling is not feasible. In light of population-based studies in children (17, 29–31) and nonpopulation-based experiments in adults (19, 25), an optimal starting design for a sophisticated super-person study might include 120 subjects, 24 sampling times from 1 h to 42 d, with all subjects sampled at a common time (e.g., 7 d) and each subject sampled at 1 additional time. For samples collected during the first day, plasma would be analyzed for both retinol and REs; retinol only would be measured in later samples. Modeling of the geometric mean RE data would provide the group mean vitamin A absorption efficiency, which would be used in the modeling of the full geometric mean retinol data set. If this analysis showed that the mean vitamin A absorption was 65%, rather than 80% as is commonly assumed in children, the model-predicted group mean TBS would be ∼20% lower than if 80% was used. Thus, including the vitamin A absorption test described here would lead to more accurate predictions of the group mean TBS as well as better values for the coefficients (since these include an estimate of vitamin A absorption); using these coefficients in an RID equation to calculate TBS for individuals based on day 7 plasma retinol specific activity would increase the accuracy of these values as well. If a third blood sample was obtained after 7 d from subjects sampled before that time, then one would obtain 2 estimates of TBS for each subject (1 at day 7 and 1 later). Application of this approach would provide reliable information on vitamin A status in individuals from the sampled population.

In summary, the results presented herein show that, by applying compartmental modeling to plasma RE kinetic data obtained during an 8-h clinical study, vitamin A absorption efficiency can be accurately measured in theoretical humans. If pilot testing shows that the method is feasible for use in healthy adults, with either the current model or a more complex one, researchers can begin to gather data on vitamin A absorption under various conditions (e.g., different levels of vitamin A status and different physiological states including those known to impact vitamin A metabolism such as inflammation and iron deficiency), leading to better recommendations for dietary vitamin A intake and more reliable assessment of vitamin A status.

## Supplementary Material

nxaa176_Supplemental_File

## Notes

Support for this work was provided by the Bill & Melinda Gates Foundation (Project Number OPP1115464).

Author disclosures: The authors report no conflicts of interest.

Supplemental Tables 1 and 2, and Supplemental WinSAAM Deck are available from the “Supplementary data” link in the online posting of the article and from the same link in the online table of contents at https://academic.oup.com/jn/.

Abbreviations used: AUC, area under the curve; RE, retinyl ester; RID, retinol isotope dilution; TBS, total body stores; t_0_, time zero; WinSAAM, Windows version of Simulation, Analysis, and Modeling software.
