# Supplementary material for: Vitamin A Absorption Efficiency Determined by Compartmental Analysis of Postprandial Plasma Retinyl Ester Kinetics in Theoretical Humans
Source: J Nutr. 2020 Jul 2;150(8):2223–9. doi: 10.1093/jn/nxaa176 (PMC7398788; doi:10.1093/jn/nxaa176)
Supplement: nxaa176_Supplemental_File [file nxaa176_supplemental_file.pdf]

Vitamin A absorption efficiency determined by compartmental analysis of postprandial plasma retinyl ester kinetics in theoretical humans. Green MH. Online Supplementary Materials

SUPPLEMENTAL TABLE 1 Assigned values for absorption efficiency, state variables, and kinetic parameters for 12 theoretical subjects[1]

Subject	1	2	3	4	5	6	7	8	9	10	11	12
Absorption (%)	55	59	64	67	70	72	74	75	77	80	86	90
State variables												
U(1), µmol/d	1.90	0.997	1.30	0.638	1.61	1.89	1.96	2.64	1.74	2.22	1.58	4.15
M(5), µmol	5	5	5	5	5	5	5	5	5	5	5	5
M(6), µmol	149	294	333	213	409	682	965	1653	786	1774	1049	1438
M(7), µmol	10.0	10.0	10.0	10.0	10.0	10.0	10.0	10.0	10.0	10.0	10.0	10.0
TBS, µmol	159	304	343	223	419	692	975	1663	796	1784	1059	1448
Kinetic parameters												
L(2,1), d^{-1}	30	30	30	30	30	30	30	30	30	30	30	30
L(0,1), d^{-1}	24.5	20.8	16.9	14.8	12.8	11.7	10.5	10.0	8.96	7.50	4.88	3.33
DT(3), d	0.0208	0.0210	0.0233	0.0278	0.0265	0.0278	0.0345	0.0235	0.0225	0.0277	0.0245	0.0208
L(15,10), d^{-1}	83.2	142	120	140	90.0	145	166	115	83.0	130	100	155
DT(15), d	0.0240	0.0200	0.0174	0.0225	0.0205	0.0243	0.0174	0.0210	0.0235	0.0195	0.0185	0.0122
L(5,4), d^{-1}	2.10	1.85	1.05	1.20	1.95	1.40	0.800	0.950	0.750	1.55	2.05	1.20
L(7,5), d^{-1}	10.00	4.00	5.00	7.00	9.00	4.50	5.50	4.00	8.00	6.00	3.00	1.00
L(5,7), d^{-1}	5.00	2.00	2.50	3.50	4.50	2.25	2.75	2.00	4.00	3.00	1.50	0.500
L(6,5), d^{-1}	2.00	6.00	5.50	2.00	3.50	3.00	2.80	8.00	4.20	11.00	3.00	6.50
L(5,6), d^{-1}	0.0600	0.100	0.0800	0.0450	0.0400	0.0200	0.0130	0.0230	0.0250	0.0300	0.0130	0.0200
L(0,6), d^{-1}	0.00700	0.00200	0.00250	0.00201	0.00275	0.00200	0.00150	0.00120	0.00170	0.00100	0.00130	0.00260

[1] Shown are assigned values for vitamin A absorption efficiency, dietary vitamin A intake [U(1)], masses [M(I)] of vitamin A in compartments 5, 6, 7, and in total body stores (compartment 6 + 7), and kinetic parameters for 12 theoretical adults. As noted in Methods, values were assigned using published values (18–20) and in light of a previously-published model (21); we assumed that vitamin A absorption was positively but not exclusively correlated with TBS (e.g., subject 12, who had the highest value for absorption, was assigned a relatively high, but not the highest, value for TBS); for other parameters, we specified a range based on published values and then randomly assigned values within that range to each subject. Kinetic parameters listed are fractional transfer coefficients [L(I,J)s, or the fraction of retinol in compartment J transferred to compartment I each day] and delay times [DT(I)s, or delay time spent in compartment I]. The model is shown in Figure 1. TBS, total body stores.

SUPPLEMENTAL TABLE 2 Assigned and model-predicted values for kinetic parameters related to vitamin A absorption in 12 theoretical subjects[1]

	Assigned	RE model	RE 5% error	Limited RE 5% error	Assigned	RE model	RE 5% error	Limited RE 5% error
Subject			1				2	
L(0,1), d^{-1}	24.5	24.5	22.5	18.8	20.8	20.7	17.9	15.0
DT(3), d	0.0208	0.0208	0.0187	0.0170	0.0210	0.0209	0.0199	0.0173
L(15,10), d^{-1}	83.2	83.6	85.3	93.4	142	143	155	162
Subject			3				4	
L(0,1), d^{-1}	16.9	16.8	20.2	20.6	14.8	14.6	11.6	12.5
DT(3), d	0.0233	0.0233	0.0236	0.0248	0.0278	0.0277	0.0271	0.0277
L(15,10), d^{-1}	120	120	113	118	140	140	158	152
Subject			5				6	
L(0,1), d^{-1}	12.8	12.8	18.6	25.3	11.7	11.5	16.5	22.2
DT(3), d	0.0265	0.0265	0.0260	0.0256	0.0278	0.0277	0.0276	0.0288
L(15,10), d^{-1}	90.0	90.1	77.8	65.0	145	146	124	106
Subject			7				8	
L(0,1), d^{-1}	10.5	10.3	10.5	11.3	10	9.97	10.2	10.1
DT(3), d	0.0345	0.0344	0.0332	0.0332	0.0235	0.0234	0.0238	0.0236
L(15,10), d^{-1}	166	167	166	166	115	115	115	114
Subject			9				10	
L(0,1), d^{-1}	8.96	9.12	8.64	12.4	7.50	7.56	7.46	9.02
DT(3), d	0.0225	0.0226	0.0197	0.0210	0.0277	0.0277	0.0266	0.0264
L(15,10), d^{-1}	83	82.6	82.3	74.5	130	130	132	124
Subject			11				12	
L(0,1), d^{-1}	4.88	4.90	4.98	6.41	3.33	3.50	3.10	3.76
DT(3), d	0.0245	0.0245	0.0229	0.0223	0.0208	0.0212	0.0208	0.0208
L(15,10), d^{-1}	100	100	100	93.6	155	155	153	145

[1] Shown are assigned values and model predictions for L(0,1), DT(3), and L(15,10) obtained by compartmental modeling of plasma RE kinetics from 30 min to 8 h (16 samples) after oral administration of labeled retinyl acetate (“RE data”), the same data sets with 5% random error added (“RE 5% error”), and a limited RE dataset (10 samples) with 5% random error added (“limited RE 5% error”) for 12 theoretical subjects. L(0,1) is equivalent to the fraction of the vitamin A dose that is not absorbed; DT(3) is the delay time in component 3; L(15,10) is related to chylomicron $t_{1/2}$. The model is shown in Figure 1 and subject details are presented in Supplemental Table 1. RE, retinyl ester.

Supplemental WinSAAM Deck

```
A SAAM31    ABS MODEL S7-M03 RE DATA 5% ERROR LIMITED [25-JAN-2020]
CC ASSIGNED ABSORPTION EFFICIENCY = 74%
CC MODEL-PREDICTED ABSORPTION EFFICIENCY = 73%
CC ASSIGNED TBS = 975 UMOL
CC PARAMETERS
H PAR
CC          VALUE          LOWER LIMIT    UPPER LIMIT
CC IC(I)=INITIAL CONDITION (FRACTION OF DOSE) IN COMPARTMENT I AT TIME 0
   IC(1)    1
CC L(I,J)=FRACTION OF J TRANSFERRED TO I PER DAY (DAY^-1)
   L(0,1)    1.126067E+01  1.181817E+00   7.363635E+01
CC L(0,1)=FRACTIONAL LOSS OF UNABSORBED TRACER
   L(3,2)=L(2,1)
CC DN(I)=NUMBER OF ELEMENTS IN DELAY COMPONENT I
CC DT(I)=DELAY TIME IN COMPONENT I (DAY)
   DT(3)     3.321835E-02  1.933333E-03   6.239999E-02
   DN(3)    8
CC OUTPUT FROM DELAY COMPONENT EQUALS 1
   L(10,3)  1
CC L(15,10)=CHYLOMICRON RE FRACTIONAL CATABOLIC RATE
   L(15,10)  1.656863E+02  2.772111E+00   2.494900E+02
H DAT
CC SIMULATED CHYLOMICRON RE DATA WITH 5% ERROR
CC                                         FRACTIONAL STANDARD DEVIATION
110                                        FSD=0.05
CC          TIME (D)      FRACION OF DOSE
            0             0
            0.02083       0
            0.04166       0.0157827
            0.06249       0.0502531
            0.08332       0.0502242
            0.12498       0.0253355
            0.16664       0.0092888
            0.2083        0.00267158
            0.24996       0.000738949
            0.29162       0.000250945
            0.33328       6.72713E-05
CC FRACTIONAL VITAMIN A ABSORPTION EFFICIENCY
111G(11)
XG(11)=(L(2,1)/(L(2,1)+L(0,1)))*100
            1
CC ABSORPTION EFFICIENCY SIMULATION
100
   G(11)
```
